# Exclusive breastfeeding status of children aged between 6 and 24 months in the nomadic population of Hadaleala district, Afar Region, northeast Ethiopia

**DOI:** 10.1186/s13006-017-0129-6

**Published:** 2017-08-24

**Authors:** Zemichael Gizaw, Wondwoson Woldu, Bikes Destaw Bitew

**Affiliations:** 1 Department of Environmental and Occupational Health and Safety, Institute of Public Health, College of Medicine and Health Sciences, University of Gondar, Gondar, Ethiopia; 2 Hadaleala District Health Office, Hadaleala District, Northeast Ethiopia, Afar Regional State Ethiopia

**Keywords:** Exclusive breastfeeding, Breastfeeding, Breastfed in first hour, Hadaleala district

## Abstract

**Background:**

Exclusive breastfeeding (EBF) during infancy is fundamental, however it is not fully practiced in the nomadic population of Ethiopia. In Ethiopia, there is still a lack of information on the implementation of the EBF, especially among the nomadic population. This study was conducted to assess the EBF status of children during their first 6 months of life, who are now aged between 6 and 24 months, in the nomadic population of Afar region. The study also aimed to identify factors affecting exclusive breastfeeding.

**Methods:**

A community based cross-sectional study was conducted from April to May, 2015 to assess EBF of children aged between 6 and 24 months during the first 6 months of life. Exclusive breastfeeding is defined as consuming only breast milk (including expressed breast milk) during the first 6 months and no other liquids and solid foods except medications, and non exclusive breastfeeding is taking liquids and solid foods in addition to breast milk. The cluster sampling technique was used to select the study participants. Data were collected from 254 households using a structured questionnaire.

**Results:**

One hundred eighty eight of the children were fed breast milk exclusively for the first 6 months of age; the rate of EBF in the study area was 74% (95% CI 70, 78%). One hundred fifty four (60.6%) of the children received breast milk within 1 h immediately after birth and 207 (81.5%) of the children maintained breastfeeding at the time of the survey. Exclusive breastfeeding was statistically associated with mothers aged above 35 years (AOR 8.3, 95% CI 1.7, 40.3), commencing to breastfeed in first hour (AOR 3.5, 95% CI 1.8, 6.9), and parents who didn’t migrate or move to a more comfortable area (AOR 4.6, 95% CI 1.5, 14.4).

**Conclusion:**

Exclusive breastfeeding was not fully practiced in the study area. Therefore, promotion of infant and young children feeding (IYCF) is needed in the area to strengthen EBF practices. Moreover, child feeding practices should be integrated with the existing health system and attention should be given to the nomadic mothers.

## Background

Breastfeeding provides an ideal food for the healthy growth and development of infants. As a global public health recommendation, infants should be exclusively breastfed for the first 6 months of life to achieve optimal growth, development and health [1, 2]. Although the benefit of exclusive breastfeeding is widely advocated globally, the rate remains low. Globally, only 40% of infants aged 6 months or less are exclusively breastfed [3], and in Ethiopia, the rate of EBF is low at 52% [4]. Ethiopian mothers practice non EBF due to different factors, such as sociodemographic [5–9], healthcare services [5–7, 9–11], and behavioral factors [6, 12–14]. While, the Ethiopian government is promoting the importance of EBF, there is still a gap with the implementation, especially among the nomadic communities. There is a lack of information on the status of EBF practices among the nomadic population. This study was conducted to assess the EBF status of children during the first 6 months of life, now aged between 6 and 24 months, in the nomadic population of Afar region. The study also aimed to identify factors associated with EBF practices.

## Methods

### Study design and settings

A community based cross-sectional study was conducted in Hadaleala district. Hadaleala district is one of the districts of Afar Regional State, which is located at 341 km southwest of the regional capital, Semera, and 268 km north of Addis Ababa, the capital city of Ethiopia. The area of 491.12 mile^2^ is divided into 11 rural kebeles (the smallest administration unit in Ethiopia) with a total population of 42,845. It has 7516 households with an average household size of 5.7 persons per house. In the district, the proportion of children aged under-5 years accounts for 10.1% (4328). The district is about 670–1280 m above sea level. The annual mean temperature of the district is 30 degree Celsius, and the district has a very variable precipitation between 440 and 760 mm annual average. Resources, like pasture and water are dispersed in the district. Due to this, the populations who live in the district are nomadic [15].

### Sample size determination and selection of study participants

The sample size was determined using the single population proportion formula by considering the following assumptions: *p* = 81% (the level of EBF among children aged less than 6 months in Amhara region) [16], 95% confidence interval, and a 5% margin of error (d),

$$ n=\frac{{\left({z}_{\frac{\alpha }{2}}\right)}^2p\left(1-p\right)}{d^2}=\frac{(1.96)^20.81\left(1-0.81\right)}{0.05^2}=237 $$

Considering a 5% non response rate, the final sample size was 249 mother-child pair.

Due to population heterogeneity across the kebeles, a cluster sampling technique was used to select the study participants. The clusters were villages with defined geographical boundaries. Six rural kebeles out of eleven were selected by the simple random sampling technique. The six targeted rural kebeles were clustered into thirty-nine villages, and seventeen villages were selected by the systematic random sampling technique. Finally, all the households who have children aged between 6 and 24 months were visited and a total of 258 study participants were included in the study.

### Outcome variable of the study

The primary outcome variable of this study was defined as an infant who was fed only breast milk (including expressed breast milk) during the first 6 months with no other liquids and solid foods except medications, and non exclusive breastfeeding defined as an infant who was fed liquids and solid foods in addition to breast milk [2, 17].

### Data collection tools and procedures

A structured questionnaire was used to collect data. The questionnaire was adapted from similar published studies [18–23] with little modification and translated to Afarigna which is the local language. To validate the adopted questionnaire, a pretest was done on children having similar characteristics with the study participants in different areas who were not part of the study. Any necessary correction was done by considering the characteristics of the participants, study settings, and other contexts. The questionnaire consists of sociodemographic and breastfeeding information. Ten diploma graduate nurses who fluently speak the local language and were working in the district were involved in the data collection process. After training on data collection, enumerators visited all the households found in the selected villages. The data collectors interviewed the mothers when they found the targeted children and ascertained the period of exclusive breastfeeding by asking the mothers when they first introduced any solid, semi-solid, or fluid to their child in addition to breast milk, in the first 6 months. For a few mothers who faced difficulties in remembering the right time of initiation of additional foods, data collectors carried out different probing mechanisms to help them to recall. Relating the time of initiation to known public events, occurrence of common childhood developmental milestones, and immunization schedules were some of the probing techniques. There was close supervision to improve the quality of the data.

### Data management and statistical analysis

Data were entered using Epi-Info version 3.5.3 statistical package and exported to SPSS version 20 for further analysis. Descriptive analysis, such as frequency, percentage or proportion, median, and interquartile ranges was conducted for most variables. Univariable binary logistic regression analysis was used to choose variables for the multivariable logistic regression analysis on the basis of *p*-value less than 0.2. Variables which had a *p*-value less than 0.2 by the univariable analysis were further analyzed by the multivariable binary logistic regression for controlling the possible effects of confounders. Statistically significant variables were identified on the basis of Adjusted Odds Ratio (AOR) with 95% Confidence Interval (CI) and *p* < 0.05.

## Results

### Sociodemographic information of children and mothers

A total of 254 mother-child pairs participated in the current study with 98.5% response rate. Half (*n* = 131, 51.6%) of the mothers were aged between 25 and 34 years. The median age of the mothers was 27 years, and the interquartile range (IQR) was 24–33 years. Almost all, 250 (98.4%) of the mothers were married at the time of the survey, and the majority 241 (94.9%) were housewives by occupation. Two hundred twenty (86.6%) of the mothers and 226 (89.0%) of the fathers didn’t attend any formal education at the time of the survey. More than half, 139 (54.7%) of the households had a maximum of five families. Of the 254 children included in this study, 133 (52.4%) were male and 158 (62.2%) were aged between 13 and 24 months; the median age was 15 months with an interquartile range (IQR) of 11 to 19 months. Forty-four (17.3%) of the children were first by their birth order, and only 31 (12.2%) of the mothers gave birth in health institutions. Nearly one-third, 82 (32.3%) of the households moved from place to place to search for suitable locations for themselves and for their livestock (Table 1).

**Table 1:** Sociodemographic information about children aged between 6 and 24 months and their mothers (*n* = 254) in Hadaleala district, Afar Region, northeast Ethiopia

Sociodemographic variables	Frequency	Percent
Age of mothe (years)		
15.0–24.0	79	31.1
25.0–34.0	131	51.6
≥ 35.0	44	17.3
Mothers’ marital status		
Currently married	250	98.4
Not currently married	4	1.6
Maternal education		
No formal education	220	86.6
Formal education	34	13.4
Maternal occupation		
Not employed	241	94.9
Employed	13	5.1
Paternal education		
No formal education	226	89.0
Formal education	28	11.0
Family size		
≤ 5	139	54.7
> 5	115	45.3
Age of children in months		
6–12 months	96	37.8
13–24 months	158	62.2
Sex of children		
Male	133	52.4
Female	121	47.6
Birth order		
First	44	17.3
Second	57	22.4
Third	54	21.3
Fourth & above	99	39.0
Place of delivery		
Home	223	87.8
Health institution	31	12.2
Migration/movement in the last 12 months		
No movement	172	67.7
Once	64	25.2
Twice	18	7.1

### Exclusive breastfeeding status of children

One hundred and fifty-four (60.6%) of the children received breast milk within 1 hour immediately after birth and the majority (*n* = 207, 81.5%) of the children maintained breastfeeding at the time of the survey. From a total of children aged between 6 and 24 months, 188 were fed breast milk exclusively during the first 6 months of age while the rest had started complementary or supplementary foods. The level of EBF practice in the area was therefore found to be 74% (95% CI 70%, 78%). The common complementary or supplementary food provided to the children was cow or goat milk, which accounted for 70.5% (*n* = 179). Two hundred thirty-three (91.7%) of the children consumed leftover foods while half (*n* = 128, 50.4%) were given uncooked foods. Nearly three-quarters of the children (*n* = 187, 73.6%) consumed foods immediately after cooking (Table 2).

**Table 2:** EBF status of children now aged between 6 and 24 months during the first 6 months of life (*n* = 254) in Hadaleala district, Afar region, northeast Ethiopia

Variables	Frequency	Percent
Breastfed in first hour		
Yes	154	60.6
No	100	39.4
Child breastfed at the time of the survey		
Yes	207	81.5
No	47	18.5
Child feeding status		
Not exclusively breastfed	66	26.0
Exclusively breastfed	188	74.0
Type of supplementary feeds		
Cow’s or goat’s milk	179	70.5
Infant formula/powder milk	9	3.5
Gruel	59	23.2
Adults’ food	7	2.8
Child consumed leftover foods		
Yes	233	91.7
No	21	8.3
Feeding children soon after food preparation		
Yes	187	73.6
No	67	26.4
Serving uncooked food/ unheated milk to children		
Yes	128	50.4
No	126	49.6

### Factors associated with breastfeeding status of children

Migration in the last 12 months, age of mothers, maternal education, occupation of mothers, marital status of mothers, paternal education, place of birth, family size, sex of children, birth order, and being breastfed in first hour were variables which fulfilled the chi-square test assumption and were entered to the univariable binary logistic regression model. Age of mothers, maternal education, paternal education, birth order, breastfed in first hour, place of birth, and migration in the last 12 months were selected for the multivariable binary logistic regression analysis. Finally, migration in the last 12 months, age of mothers, and breastfed in first hour were significantly associated with exclusive breastfeeding status in the multivariable binary logistic regression analysis (Table 3).

**Table 3:** Factors associated with EBF status of children now aged between 6 and 24 months, during the first 6 months of age in Hadaleala district, Afar Region, northeast Ethiopia

Variables	Breastfeeding status		COR (95% CI)	AOR (95% CI)
	Exclusively breastfed (in number)	Not exclusively breastfed (in number)		
Place of birth				
Home	160	63	1	
Health institution	28	3	3.7 (1.1, 12.5)	0.8 (0.2, 3.7)
Age of mothers in year				
15.0–24.0	56	23	1	
25.0–34.0	92	39	1.0 (0.5, 1.8)	1.9 (0.7, 5.7)
≥ 35.0	40	4	4.1 (1.3, 12.8)	8.3 (1.7, 40.3)**
Maternal education				
No formal education	157	63	1	
Formal education	31	3	4.1 (1.2, 14.1)	1.4 (0.3, 6.3)
Paternal education				
No formal education	161	65	1	
Formal education	27	1	10.9 (1.5, 81.9)	4.9 (0.5, 44.6)
Breastfed in first hour				
Yes	132	22	4.7 (2.6, 8.6)	3.5 (1.8, 6.9)***
No	56	44	1	
Migration during the last year				
No movement	134	38	7.1 (2.5, 20.0)	4.6 (1.5, 14.4)*
Once a year	48	16	6.0 (1.9, 18.6)	4.4 (1.3, 14.8)*
Twice a year	6	12	1	
Birth order				
1st	38	6	2.1 (0.8, 5.7)	3.5 (0.8, 15.4)
2nd	39	18	0.7 (0.4, 1.5)	1.1 (0.4, 3.4)
3rd	37	17	0.7 (0.4, 1.5)	1.1 (0.5, 2.7)
4th and above	74	25	1	

* Statistically significant variables at *p* < 0.05, ** statistically significant variables at *p* < 0.01, *** statistically significant variables at *p* < 0.001, the result of Hosmer and Lemshow test was >0.470

This study indicated that the mother’s age was statistically associated with breastfeeding status of children for the first 6 months of age. The odds of practicing EBF was 8.3 times more likely to be higher amongst mothers aged above 35 years compared with mothers aged between 15 and 24 years (AOR 8.3, 95% CI 1.7, 40.3). Breastfeeding in the first hour was found to be associated with the practice of EBF. Exclusive breastfeeding was 3.5 times more likely to be higher among mothers who initiated breastfeeding within 1 hour immediately after birth compared with their counterparts (AOR 3.5, 95% CI 1.8, 6.9). Moreover, displacement or instability was the other variable associated with EBF; EBF was 4.6 times more likely to be higher among children whose parents didn’t migrate from place to place searching for suitable locations for themselves and their livestock, compared with families who migrated twice a year (AOR 4.6, 95% CI 1.5, 14.4). The practice of EBF was also associated with frequency of migration. For instance, the practice of EBF was 4.4 times higher among children whose parents migrated once a year compared with children whose parents moved twice a year (AOR 4.4, 95% CI 1.3, 14.8).

## Discussion

This study investigated the exclusive breastfeeding status of children during the first 6 months of life, when aged between 6 and 24 months. The level of EBF practice in the area was found to be 74% (95% CI 70, 78%). The level of EBF reported in this study is consistent with findings of other similar studies conducted in Ethiopia, for instance studies conducted in Kersa district (71.7%) [7] and in Halaba special district (70.5%) [9]. EBF in the current study is higher than the 2011 national prevalence (52%) [4], and the findings of studies conducted in Ankesha Guagusa District (53%) [24], Bahir Dar District (50.3%) [18], Dabat district (30.7%) [25], and Axum (40.9%) [26]. This may be due to the small population size in the study area and that health extension workers can frequently provide health information and other healthcare services to mothers. Moreover, most of the mothers in the study area spent their time at home, which could increase the mother’s time to provide complete care. Other studies have confirmed that work outside the home is a primary barrier to EBF [27, 28].EBF was higher in studies conducted in Egypt (97.7%) [29], and Ghana (79%) [30], which might be due to differences in healthcare coverage and accessibility.

Of the 24 % of children in this study who had started supplementary or complementary foods, cow or goat milk (70.5%), gruel (23.2%), infant formula/powdered milk (3.5%), and adults’ food (2.8%), were the common types of complementary or supplementary foods. Almost all (91.7%) of the children consumed leftover foods and 26.4% of the mothers didn’t provide foods immediately after preparation. Moreover, 50.4% consumed uncooked foods. These conditions might increase the risks of food borne infections, especially in regions where sanitation conditions are poor. Early intake of unhygienic complementary foods reduces the intake of breast milk and as a consequence, infants receive fewer protective factors.

This study indicated that a mothers’ age was associated with the breastfeeding status of children in the first 6 months of life. Older mothers were more likely to practice EBF than the younger mothers. Other similar studies had also reported similar findings [5, 31–34]. Older mothers might have more experience and knowledge of the health importance of exclusive breastfeeding. Moreover, older mothers might have more antenatal and postnatal care, as well greater knowledge about child care.

Similar to other studies [9, 22, 29, 35–40], this study showed that mothers’ EBF practice was associated with early or timely breastfeeding inititation. Mothers who initiated breastfeeding immediately after birth were more likely to practice EBF than mothers who did not. This might be due to the fact that mothers who initiated breastfeeding within 1 hour immediately after birth may understand the nutrient, growth, and the immunologic importance of breastfeeding. As a result, mothers could maintain breastfeeding. The World Health Organization (WHO) report also shows that breastfed in first hour increases the likelihood of exclusive breastfeeding for one to 4 months of life, as well as the overall duration of breastfeeding [41].

This study also explored whether displacement or instability of households was associated with EBF. Mothers who did not move or migrate from place to place were more likely to practice EBF than mothers who were displaced or migrated. This might be due to the fact that nomadic populations move from place to place to search for more suitable places for their livestock. Most of the livestock give birth when they are in a comfortable place and the nomads intend to give animal milk to the children [42–45]. Furthermore, displaced mothers may not have time to give care for children because they may be very busy to resettle themselves and to search drinking water sources.

## Conclusion

Exclusive breastfeeding was not fully practiced in the study area. Therefore, promotion of infant and young children feeding (IYCF) is needed in the area to strengthen EBF practices. Moreover, attention should be given to the nomadic mothers and child feeding practices should be integrated with the existed health system.

## Abbreviations

AOR: Adjusted odds ratio
CI: Confidence interval
COR: Crude odds ratio
EBF: Exclusive breastfeeding
HDS: Health and demographic survey
IQR: Interquartile range
IYCF: Infant and young child feeding
Km: Kilometer
mm: Millimeter
SPSS: Statistical package for social sciences
UNICEF: United Nations Children’s Fund
WHO: World Health Organization
